# Supplementary material for: Defining Mitochondrial Cristae Morphology Changes Induced by Aging in Brown Adipose Tissue
Source: Adv Biol (Weinh). Author manuscript; Available in PMC 2024 Feb 16. (PMC10869235; doi:10.1002/adbi.202300186)
Supplement: suppl. figure 1-3 [file NIHMS1962717-supplement-suppl__figure_1-3.pdf]

Supplemental Figure 1: Display of raw mitochondrial quantifications. Each bar represents one of the three mice surveyed at respective age points while dots represent individual mitochondria (n is approximately 300). Values are displayed for (A) mitochondrial volume, (B) area, (C) perimeter, (D) complexity index, and (E) sphericity.

Supplemental Figure 2: Display of raw cristae quantifications. Each bar represents one of the three mice surveyed at respective age points while dots represent individual mitochondria (n is approximately 300). Values are displayed for (A) cristae volume, (B) area, and (C) perimeter.

Supplemental Figure 3: Overall cristae-otyping changes across aging. (A) 3-month murine BAT 3D reconstruction of cristae viewed from the transverse and (A') 3D reconstruction viewed from a longitudinal point of view. (B) 2-year murine BAT 3D reconstruction of cristae viewed from the transverse point of view (B') 3D reconstruction viewed from a longitudinal point of view. (C) Cristae-otyping to compare cristae morphology in samples from 3-month and 2-year across volumes.

SFig. 1

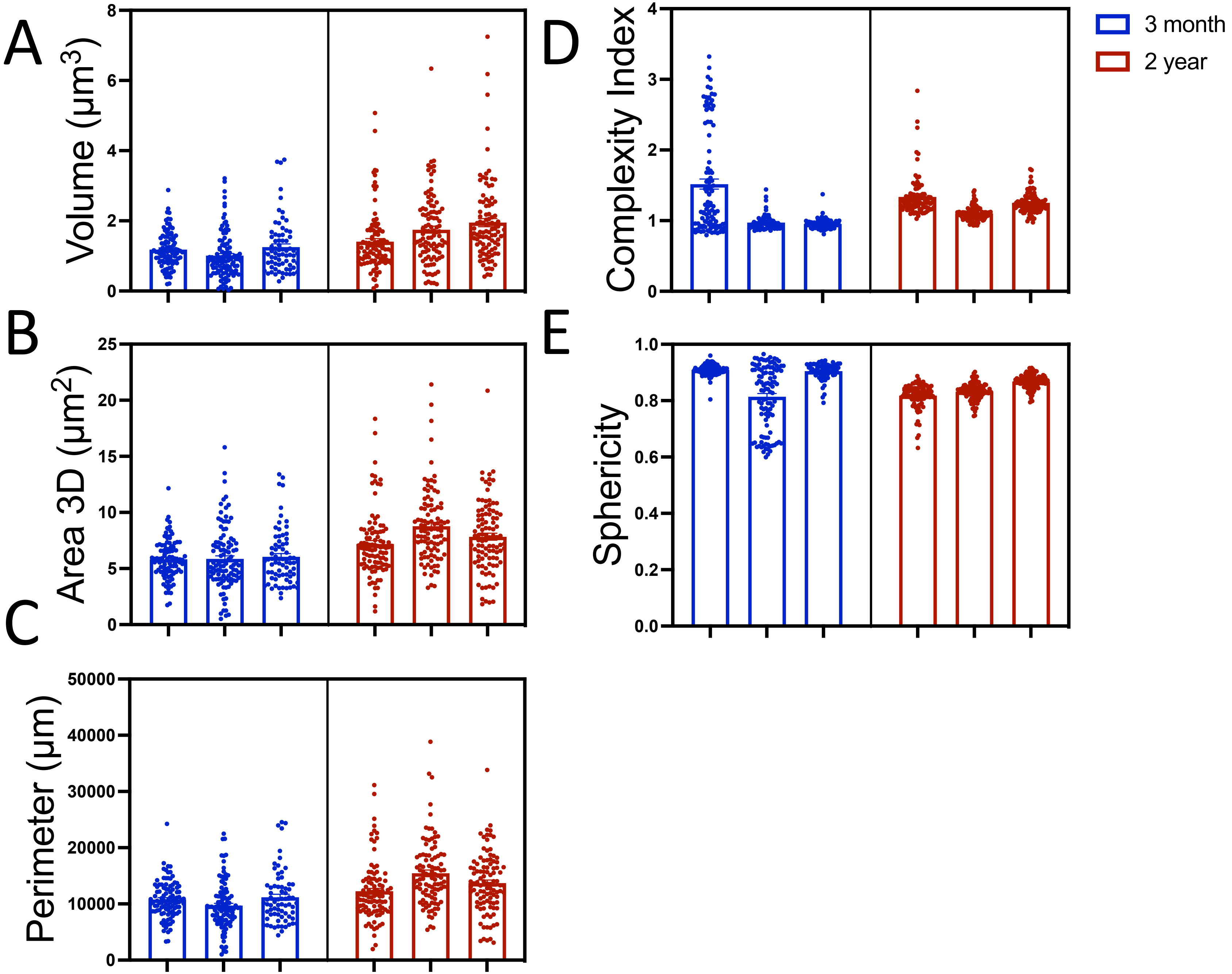

A
B
C
D
E
3 month
2 year
Volume (μm3)
8
6
4
2
0
Area 3D (μm2)
25
20
15
10
5
0
Perimeter (μm)
50000
40000
30000
20000
10000
0
Complexity Index
4
3
2
1
0
Sphericity
1.0
0.8
0.6
0.4
0.2
0.0

SFig. 2

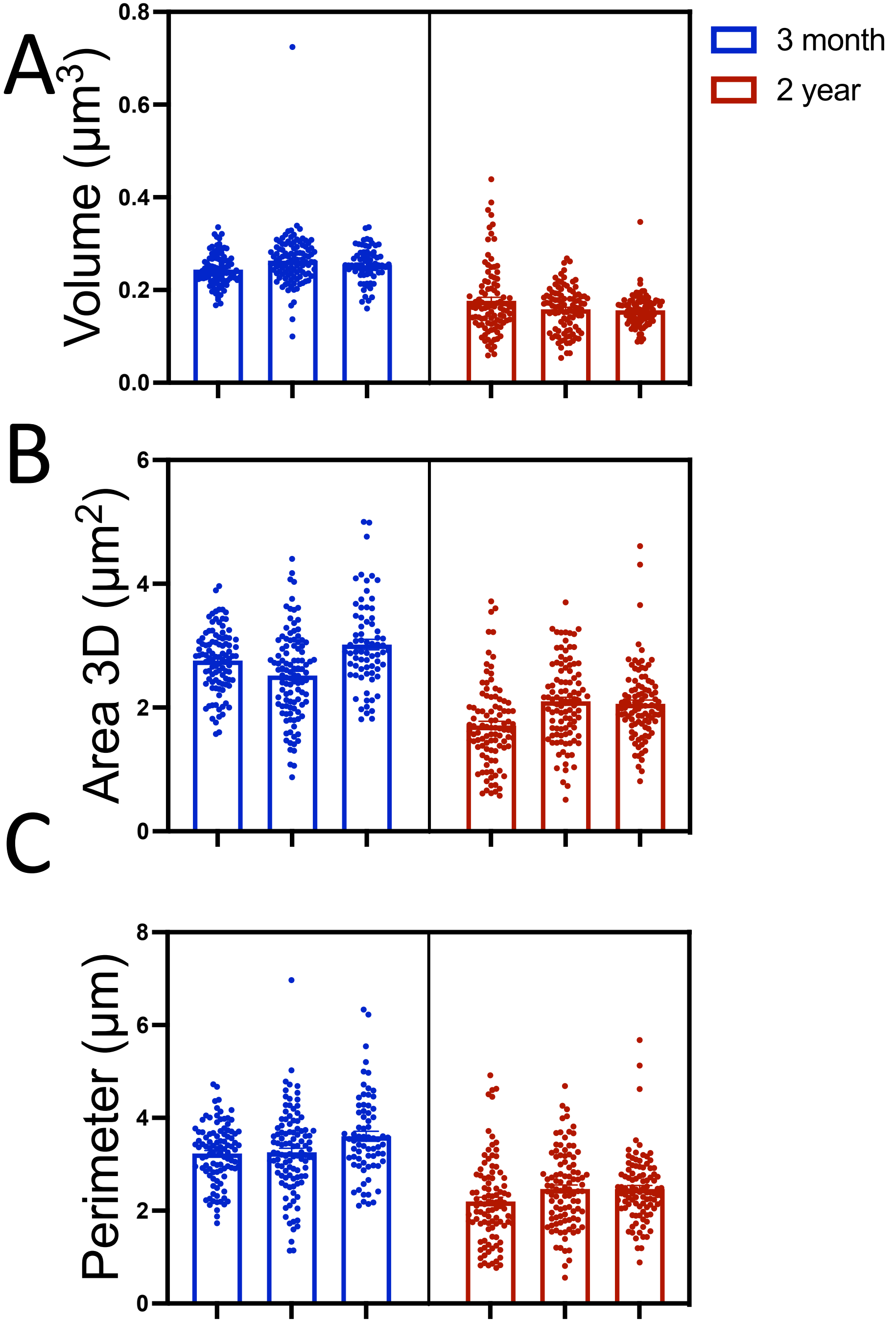
A
B
C
3 month
2 year
Volume (µm3)
0.0
0.2
0.4
0.6
0.8
Area 3D (µm2)
0
2
4
6
Perimeter (µm)
0
2
4
6
8

SFig. 3

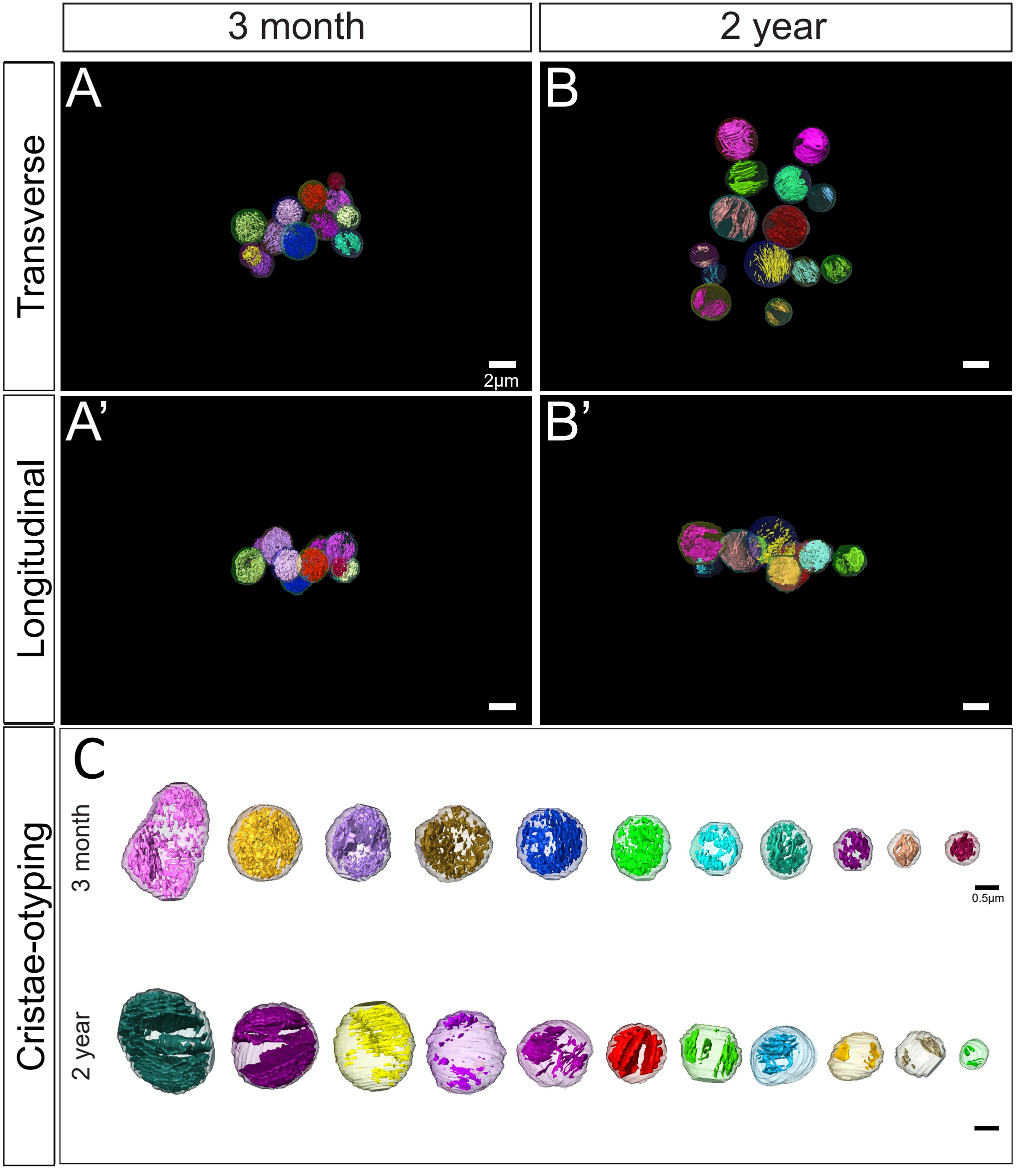
3 month
2 year
Transverse
Longitudinal
Cristae-otyping
A
B
A'
B'
C
2μm
3 month
2 year
0.5μm
